# Neural Processing of Dynamic Animated Social Interactions in Young Children With Autism Spectrum Disorder: A High-Density Electroencephalography Study

**DOI:** 10.3389/fpsyt.2019.00582

**Published:** 2019-08-22

**Authors:** Reem K. Jan, Tonia A. Rihs, Nada Kojovic, Holger F. Sperdin, Martina Franchini, Anna Custo, Miralena I. Tomescu, Christoph M. Michel, Marie Schaer

**Affiliations:** ^1^College of Medicine, Mohammed Bin Rashid University of Medicine and Health Sciences, Dubai, United Arab Emirates; ^2^Developmental Imaging and Psychopathology Lab, Department of Psychiatry, University of Geneva, Geneva, Switzerland; ^3^Functional Brain Mapping Laboratory, Department of Fundamental Neuroscience, University Medical School, Geneva, Switzerland

**Keywords:** ASD, high-density EEG, source imaging, eye-tracking, frontal, cingulate, parietal, cerebellum

## Abstract

**Background:** Atypical neural processing of social visual information contributes to impaired social cognition in autism spectrum disorder. However, evidence for early developmental alterations in neural processing of social contingencies is scarce. Most studies in the literature have been conducted in older children and adults. Here, we aimed to investigate alterations in neural processing of social visual information in children with autism spectrum disorder compared to age-matched typically developing peers.

**Methods:** We used a combination of 129-channel electroencephalography and high-resolution eye-tracking to study differences in the neural processing of dynamic cartoons containing human-like social interactions between 14 male children with autism spectrum disorder and 14 typically developing male children, aged 2–5 years. Using a microstate approach, we identified four prototypical maps in both groups and compared the temporal characteristics and inverse solutions (activation of neural sources) of these maps between groups.

**Results:** Inverse solutions of the group maps that were most dominant during free viewing of the dynamic cartoons indicated decreased prefrontal and cingulate activation, impaired activation of the premotor cortex, and increased activation of parietal, temporal, occipital, and cerebellar regions in children with autism spectrum disorder compared to their typically developing peers.

**Conclusions:** Our findings suggest that impairments in brain regions involved in processing social contingencies embedded in dynamic cartoons are present from an early age in autism spectrum disorder. To the best of our knowledge, this is the first study to investigate neural processing of social interactions of children with autism spectrum disorder using dynamic semi-naturalistic stimuli.

## Introduction

Autism spectrum disorder (ASD) is a heterogeneous neurodevelopmental disorder characterized by social and communication deficits, repetitive behaviors, and restricted interests, with a prevalence of approximately 1 in 59 in the US (1). ASD is currently defined as a single entity by the *Diagnostic and Statistical Manual of Mental Disorders* (*DSM-5*) (2), where the term spectrum refers to the heterogeneity in the range and severity of symptoms among others.

Despite the heterogeneity in ASD symptoms, deficits in social cognition have consistently been reported in individuals on the spectrum at all ages and have been suggested to represent a core deficit in ASD (3). These deficits start from a very early age and can lead to inadequate social experiences required for “social learning,” as well as insufficient “cognitive learning” (4, 5). Individuals with ASD generally exhibit abnormalities in eye contact and the processing of biological motion and facial information (6, 7), and struggle attributing social meaning to visual stimuli when they are ambiguous (8) compared to their typically developing (TD) peers. As such, deficits in orienting to people and the resultant reduction in or lack of social interactions may be a hallmark of autism (9).

As the child develops, these insufficiencies in social cognition are thought to result in impaired development of brain regions responsible for processing social information, and impaired cognitive development, such as the fusiform gyrus (FG), amygdala, superior temporal cortex, anterior temporal cortex, temporo-parietal junction, medial prefrontal cortex, anterior cingulate cortex (ACC), precuneus, inferior frontal cortex, and inferior parietal lobule (IPL) [for reviews, see Frith (10), Pelphrey et al. (3), and Schaer et al. (11)].

Neuroimaging studies have revealed a different organization and functioning at the large-scale brain level in ASD. Functional studies of social cognition have, for example, highlighted robust differences in activation of these regions between individuals with ASD and their TD peers (12–14). Structural abnormalities have also been found in several of these brain regions including reduced grey matter in the ACC and temporal cortex of individuals with ASD aged 8–50 years (15), and in the temporal cortex and IPL of adults with ASD (16, 17). Network abnormalities have also been reported in several brain regions including the frontal lobes (18). However, to date, most functional brain studies using social stimuli have been conducted in school-aged children, adolescents and adults, whereas putative deficits in neural processing of social cognition in toddlers and preschoolers, at the time of ASD diagnosis, have been poorly examined.

Eye-tracking is a powerful tool for measuring the social component of visual perception and has been used to quantitatively confirm the clinically observed reduction of interest in social cues and interactions in individuals with ASD (19–21). Another powerful tool for studying social cognition is high-density electroencephalography (EEG), which is used to study the brain’s electro-cortical activity at a large-scale level. It is particularly useful in infants and children because it is non-invasive, can be informative regardless of communication ability, and requires less physical adjustment to the equipment in comparison to other neuroimaging techniques such as magnetic resonance imaging (22, 23). EEG studies have shown that an abnormal pattern of brain activity in response to faces versus objects is present early in life in ASD (24–26) and that this abnormal pattern is also present in 10-month-old infants at risk for autism, suggesting that abnormality in face versus object processing is an early indicator of risk for developing ASD (27). Recently, by combining high-density EEG and eye-tracking, we found that directed functional connectivity alterations of social brain networks is a core component of atypical brain development at early stages of ASD (28, 29).

Here, we used a combination of high-density EEG and eye-tracking in children with ASD aged 2–5 years and compared them to age-matched TD children, to investigate alterations in neural processing of semi-naturalistic cartoons that explicitly depict human-like social interactions. Analysis of the EEG data was performed using a microstate analysis technique, which is a data-driven, reference-free approach [for a review, see Michel and Koenig (30)]; EEG microstates are short-lasting (∼100 ms) periods of stable topographies of the electric potentials in the ongoing EEG (31). Typically, only a few archetypical maps represent the majority of the broad-band resting EEG, reproducible within and across subjects (32). EEG microstate analysis thus allows the parsing of the ongoing broad-band EEG into a limited number of distinct quasi-stable states, reflecting short-lasting coordinated activation of large-scale brain networks, continuously alternating between each other (33, 34). The analysis of the temporal dynamics of the microstate time series, their individual presence and duration, as well as their source localization offers a new way of looking at brain network dynamics in the ongoing EEG during different mental and cognitive states (30, 35–37).

We hypothesized that children with ASD would exhibit differences in activation of brain regions that are typically specialized in social information processing compared to their TD peers, such as, the FG, amygdala, superior temporal cortex, anterior temporal cortex, temporo-parietal junction, medial prefrontal cortex, ACC, precuneus, inferior frontal cortex, and IPL. Subsequently, we also used eye-tracking gaze data to divide the ASD group according to their gaze behavior, to conduct an exploratory subgroup analysis which aimed to investigate whether autistic children with control-similar (CS) gaze patterns (CS ASD) showed significant differences in the way their brain processed social information from children with control-dissimilar (CD) gaze patterns (CD ASD). We hypothesized that activation of the abovementioned regions, involved in social information processing, would be affected by visual exploration patterns of children with ASD, with greater differences in neural activation expected between CD ASD and TD than between CS ASD and TD children. Although this exploratory subgroup analysis was expected to yield interesting results, these were considered to be preliminary due to the small sample size of the ASD subgroups. Hence, interpretation of the results from the subgroup analysis was exercised with caution, and future studies of larger sample size are recommended for consolidation of results.

## Methods

### Participants

High-density EEG and eye-tracking data were successfully collected from 46 children aged 2–5 years, of whom 21 had a confirmed diagnosis of ASD (2 females) and 25 were TD (10 females). Given the well-documented gender bias in ASD (38, 39), and that we only had EEG data from 2 females with ASD, this paper solely investigated male participants, and data from female participants (ASD = 2, TD = 10) were excluded from the analysis, resulting in 19 ASD and 15 TD male children. Following data inspection, five participants with ASD and one participant with TD were excluded due to unrepairable noisy signal. Thus, the final group included in the EEG analysis was composed of 14 participants with ASD (mean age 3.3 ± 0.8 years) and 14 age-matched TD participants (mean age 3.2 ± 0.9 years).

Participants with ASD were recruited through French-speaking parent associations and specialized clinical centers. All participants in the ASD group had received a clinical diagnosis of ASD prior to their inclusion in the research protocol, and none of them had any known neurogenetic conditions such as Fragile X, Rett, or Phelan McDermid syndromes, or neurofibromatosis. As a part of the research protocol, diagnosis was confirmed using either the Autism Diagnostic Observation Schedule—Generic (ADOS-G) (40) or the Autism Diagnostic Observation Schedule, second edition (ADOS-2), the latter including a toddler module that defines concern for ASD (41). ADOS assessments were administered and scored by trained psychologists who met requirements for research reliability. ADOS-G scores were then transformed according to Gotham and colleagues’ algorithm (42), and ADOS-2 toddler module scores were transformed into standardized calibrated severity scores according to Esler and colleagues’ method (43), to facilitate comparison of scores from different modules. On a scale of 10, the mean severity score for the ASD group in the current study was 7.4 ± 1.9.

TD participants were recruited through announcements in the Geneva community. All TD participants were screened for neurological/psychiatric deficits and learning disabilities and family history of ASD prior to their inclusion in the research protocol. For all participants, a telephone interview and a medical development history questionnaire were conducted prior to their first visit to the research center. TD children also underwent ADOS-G or ADOS-2 evaluations in order to ensure typical development and exclude any signs of ASD. All TD children had a minimal ADOS severity score of 1, except for one child who had a score of 3 but did not belong on the spectrum, since scores of 1–3 receive a non-spectrum ADOS classification (42). Researchers ensured that the parents or legal guardians of participants understood the study protocol and gave their informed consent for participation and publication of results prior to their inclusion in the study. The study protocol conforms with the Code of Ethics of the World Medical Association (Declaration of Helsinki) and was approved by the Local Research Committee, the Commission Centrale d’Ethique de Recherche (CCER) in Geneva, Switzerland.

### Stimuli and Procedure

**Figure 1:**
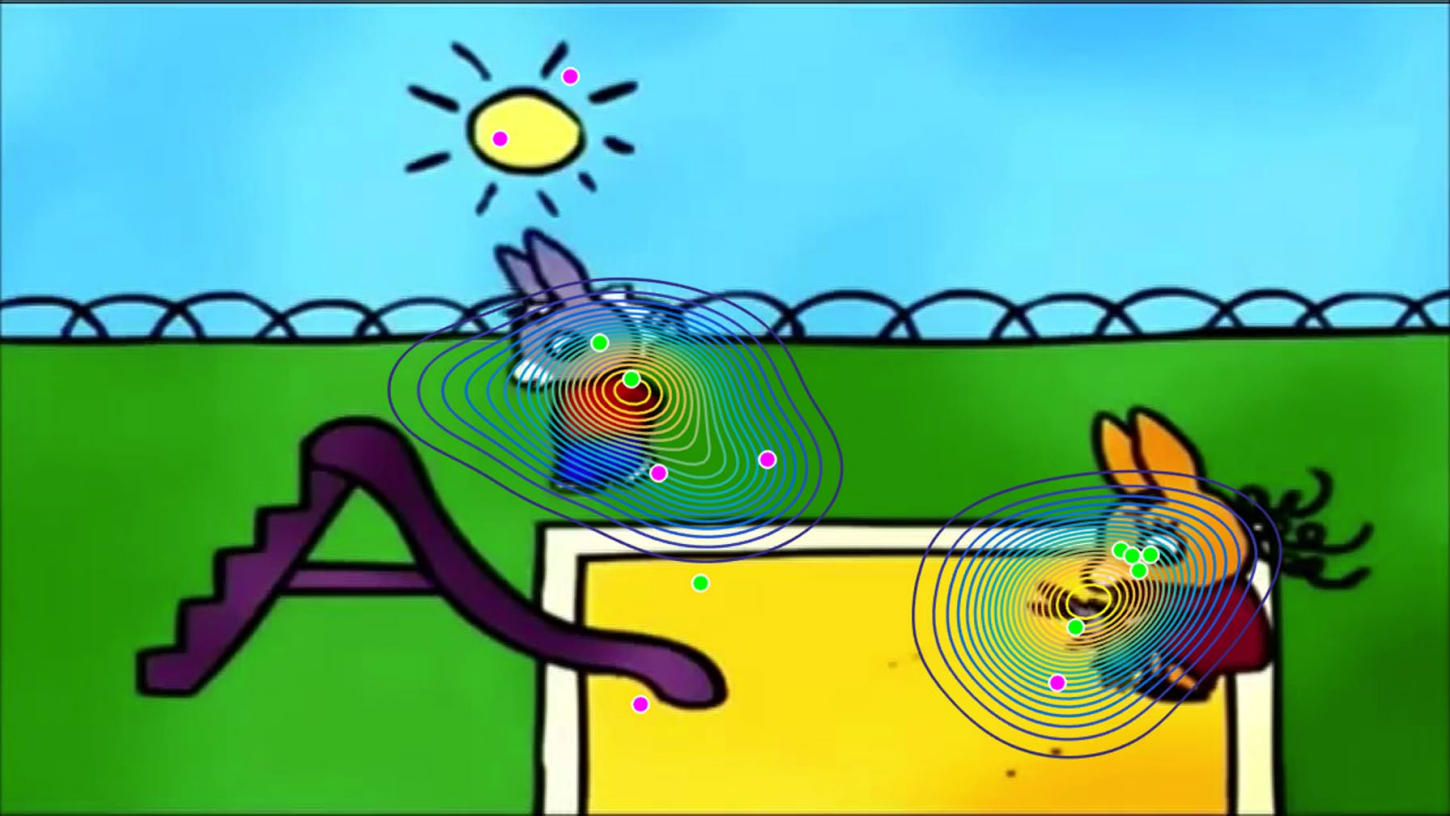
A video still from a “Trotro” cartoon film illustrating the position of the “norm” in gaze, which was established using a kernel density distribution estimation on gaze data from a group of 26 typically developing children aged 2–5 years (represented here with contour plots). For this particular frame, the gaze of typically developing children was split into two foci of interest, centered on the main characters. The gaze of each child with autism spectrum disorder (ASD) in this sample is demonstrated with individual dots. Green dots represent gaze coordinates of control-similar (CS) children with ASD (n = 8), while pink dots represent gaze coordinates of control-dissimilar (CD) children with ASD (n = 6). It is important to note that the subgrouping into CS and CD children with ASD is based on average gaze data from the entire movie. Therefore, it follows that for this specific frame, two CD ASD children, whose gaze patterns were, on average, far from the “norm” over the course of the movie, were focusing closer to the center of attention of TD children than one CS ASD child.

The stimulus of interest in this study was an animated cartoon movie named *Trotro*, which depicts the donkey Trotro engaging in human-like social interactions with his parents or friends or playing with toys across various scenes using both body language and spoken French (44) (**Figure 1**). Presentation of stimuli was controlled by Tobii Studio software v. 3.2 (Tobii^®^ Technology, Sweden). Participants were seated approximately 65 cm away from the recording screen. A five-point calibration was performed using a built-in Tobii mechanism using child-friendly animations. The testing room had no windows, and lighting conditions were kept constant. All participants watched four 2- to 3-min movies of the Trotro animated cartoon. The movies were organized in two blocks, separated by a short break. The stimuli of interest (Trotro) were interspersed with another set of stimuli that were intended to analyze biological and non-biological motion processing. All participants watched the same movies in the following order: Block 1: 1) Trotro movie 1: Trotro amoureux (Trotro is in love); 2) non-biological motion (part 1); 3) Trotro movie 2: Trotro et la boite des secrets (Trotro and the box of tricks); 4) biological motion (part 1). Block 2: 5) Trotro movie 3: Trotro part en vacances (Trotro goes on holidays); 6) non-biological motion (part 2); 7) Trotro movie 4: L’anniversaire de Nana (Nana’s birthday); 8) biological motion (part 2). Here, we investigated data collected during the four Trotro movies, with a total duration of 11 min 11 s. The data concerning biological and non-biological motion stimuli will be published separately.

### EEG Data Collection and Analysis

#### EEG Data Acquisition and Pre-processing

High-density EEG was continuously recorded with a 129-channel Hydrocel Geodesic Sensor Net^®^ (Electrical Geodesics Inc., Eugene, OR, USA). The EEG data were acquired with a sampling rate of 1,000 Hz and a recording reference at the vertex; impedances were kept below 30 kΩ. Electrodes located on the cheeks and on the nape were excluded, and 110 electrodes were retained for further analysis, which was performed using the free academic Cartool software v. 3.60 (http://sites.google.com/site/cartoolcommunity, Geneva, Switzerland) (45). The two runs of EEG data were concatenated and digitally filtered between 1 and 40 Hz, using a second-order Butterworth filter with a −12 db/octave roll-off. The filter was computed linearly with two passes, one forward and one backward, in order to eliminate phase shifts, and with poles calculated each time to the desired cut-off frequency. Subsequently, an additional notch filter was applied to eliminate 50 Hz noise. Data contaminated by oculomotor artifacts were excluded using an infomax independent component analysis implemented in Matlab (46–48). For each participant, channels exhibiting substantial noise were interpolated using a 3-D spline interpolation procedure (49). On average, 15.5 channels were interpolated for each participant. Data were re-referenced to the common average reference, downsampled from 1,000 to 125 Hz, and reduced to the local maxima of the global field power (GFP), in order to improve signal-to-noise ratio (33, 45, 50).

#### EEG Microstate and Source Analyses

For each participant, the topographic maps at GFP peaks from the four Trotro cartoon conditions were submitted to a k-means spatial cluster analysis (45, 50, 51), to identify templates of the most dominant topographic maps present during free viewing of Trotro cartoons, using a weighted optimum of 11 selection criteria (52). Only segments that were free of muscular artifacts as identified by visual inspection were considered for the k-means analysis. Following the individual-level cluster analysis, the dominant topographic maps from each subject were submitted to a second group-level k-means cluster analysis, which identified the most dominant topographic maps (the microstate maps) for each participant group. To assess whether the microstate maps from each group differed topographically, an unpaired topographic analysis of variance (TANOVA) was conducted for each pair of maps (e.g. ASD Map 1 and TD Map 1) (53), and a Pearson’s spatial correlation analysis was carried out on the two groups of maps.

In order to estimate the temporal characteristics of the dominant maps within each group, spatial correlation was conducted between the microstate maps and the actual topographic maps at each time point of each participant’s artifact-corrected EEG data from the Trotro cartoon conditions. This resulted in each participant’s data at each time point being assigned to one of the microstate maps with which it correlated best (45, 51). To ensure that data segments were not artificially interrupted by noise during low GFP, temporal smoothing was conducted with a window half size of 3 and strength (Besag factor) of 10 (45, 50). The labeling process allowed for the computation of the following temporal parameters of each of the microstate maps: global explained variance (GEV), mean duration, time coverage, and frequency of occurrence. The GEV is an estimate of the explained variance of a given map, weighted by the GFP. The mean duration is the average duration (in ms) of EEG data segments that were assigned to a given group map, whereas the time coverage is the percentage of total time in individual EEG data that is represented by a given microstate map. The frequency of occurrence represents the number of times per second that a given microstate map occurs in the individual EEG data. In order to determine the most dominant maps within each group and compare these between groups, a one-way analysis of variance (ANOVA) with temporal parameters as the dependent variables and group map (1, … n) as the within-subjects factor was performed using Statistica software v. 13 (Dell Inc., Tulsa, OK, USA). The results and post hoc tests were Bonferroni-corrected for multiple comparisons.

For source localization, all time points that were assigned to a given microstate map were concatenated for each subject. A linear distributed inverse solutions was then computed for each time point using the Local Autoregressive Average (LAURA) regularization approach, described in Grave de Peralta et al. (54) and Grave de Peralta et al. (55), and implemented in Cartool software (45). Five thousand solution points were constrained to and equally distributed in the grey matter using the Montreal Neurological Institute (MNI) template brain for toddlers aged 33–44 months with consideration of skull thickness (Locally Spherical Model with Anatomical Constraints, LSMAC) (56–56). The source maps were then averaged across all time points for each subject and for each microstate map separately (59).

In order to calculate differences in neural activation between ASD and TD, the sources underlying the microstate maps that had the highest temporal parameters were compared using a non-parametric unpaired randomization test with an exhaustive evaluation of all possible permutations of the subjects (more than 16,000 iterations) (60). The probability threshold of this randomization test was set to *P* < 0.01. In order to determine the direction of the difference (i.e. ASD > TD vs. TD> ASD), unpaired t-tests were performed, and the t-values were thresholded to the *P*-values of the permutation test (*P* < 0.01). Brain regions with significant differences in activation between the two groups were identified by consensus labeling by researchers trained in neuroanatomy.

## Results

### Dominant Group Maps

**Figure 2:**
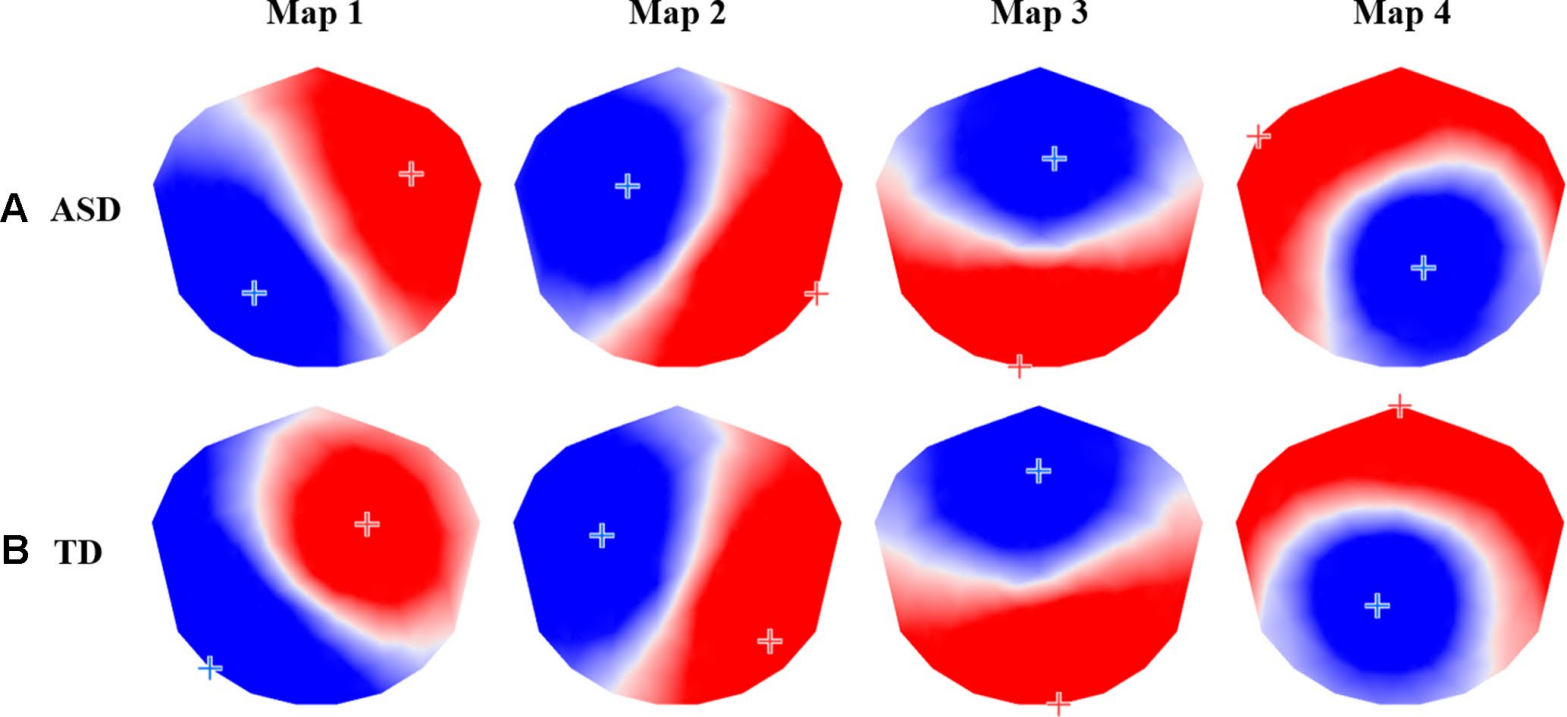
The four prototypic maps identified by the group-level k-means cluster analysis for **(A)** autism spectrum disorder (ASD) and **(B)** typically developing (TD) participants.

The group-level k-means cluster analysis identified four dominant topographic maps for each participant group, which explained 80.44% of the total variance for the ASD group and 77.43% of the total variance for the TD group (**Figure 2**). A Pearson’s spatial correlation analysis revealed that each of the four dominant microstate maps for the ASD group was highly correlated with one of the microstate maps for the TD group. TANOVA analysis showed no significant differences in topography of the corresponding maps between groups (*P* > 0.05), and (**Table 1**).

**Table 1:** Pearson’s spatial correlation coefficients between autism spectrum disorder (ASD) and typically developing (TD) group map pairs.

	TD Map 1	TD Map 2	TD Map 3	TD Map 4
**ASD Map 1**	0.88*	0.56*	0.40*	0.58*
**ASD Map 2**	0.22	0.99*	0.64*	0.01 NS
**ASD Map 3**	0.66*	0.37*	0.98*	0.52*
**ASD Map 4**	0.16 NS	0.50*	0.61*	0.84*

*Correlations significant at P < 0.003 (Bonferroni-adjusted), NS: P > 0.05.

### Temporal Dynamics

**Figure 3:**
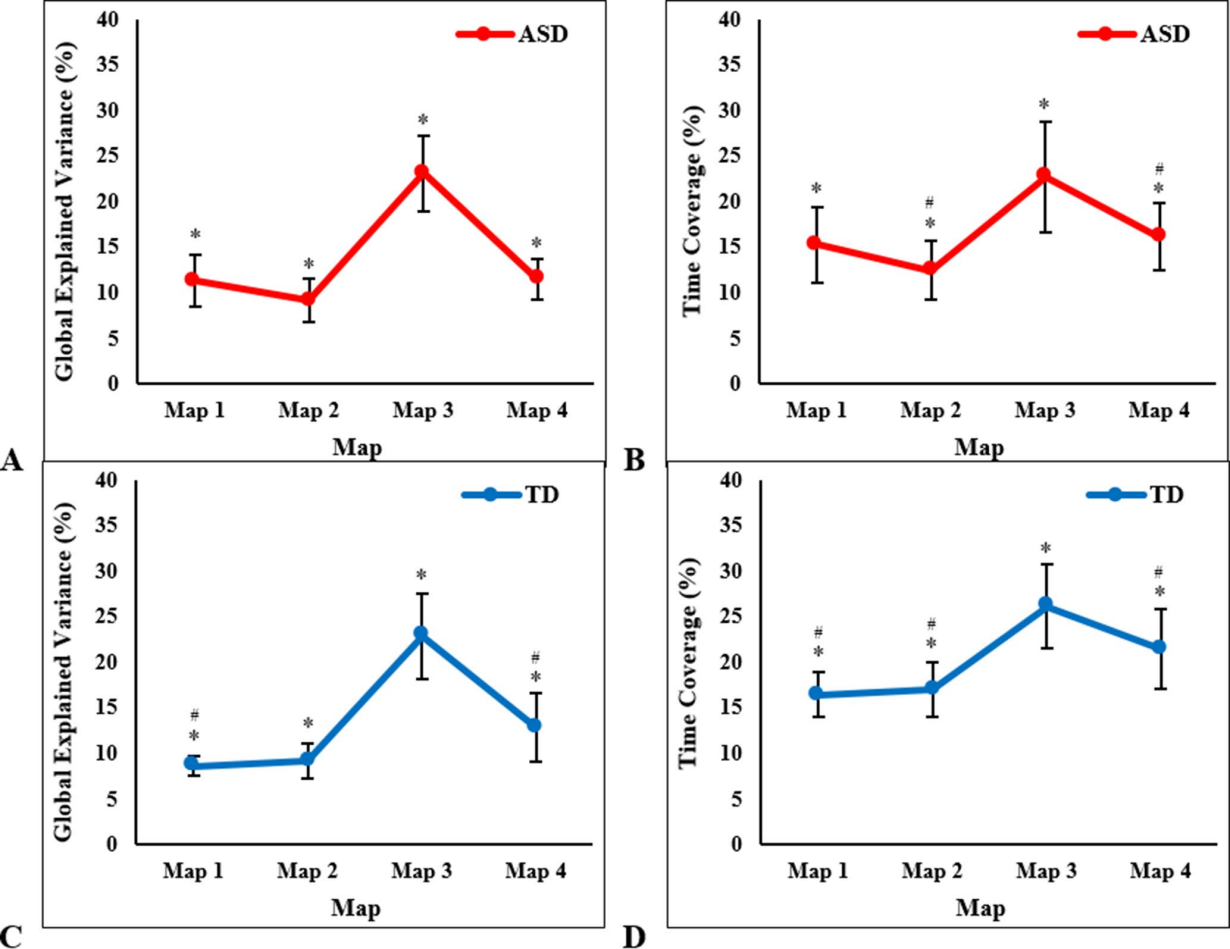
One-way ANOVA results showing significant between-map differences in children with autism spectrum disorder (ASD), in mean (± SD): **(A)** global explained variance and **(B)** time coverage; and in typically developing (TD) children, in mean (± SD): **(C)** global explained variance and **(D)** time coverage. *Significant *post hoc* tests with Map 3, ^#^significant *post hoc* tests with Map 4, at *P* < 0.003 (Bonferroni-adjusted).

Within each participant group, a one-way ANOVA with temporal parameters as the dependent variables and microstate map (1, 2, 3, 4) as the within-subjects factor revealed significant differences in each of the four temporal parameters between maps. For the ASD group, there were significant differences between maps in GEV (F_3, 39_ = 50.22, *P* < 0.001, **Figure 3A**), mean duration (F_3, 39_ = 31.58, *P* < 0.001), time coverage (F_3, 39_ = 26.36, *P* < 0.001, **Figure 3B**), and frequency of occurrence (F_3, 39_ = 21.40, *P* < 0.001). Bonferroni-adjusted post hoc tests revealed that Map 3 had significantly higher temporal parameters than all other maps (*P* < 0.001), and Map 4 had higher mean duration (*P* = 0.007), time coverage (*P* = 0.02), and frequency of occurrence (*P* = 0.02) than Map 2. Within the TD group, Map 3 had the highest GEV (F_3, 39_ = 46.06, *P* < 0.001, **Figure 3C**), mean duration (F_3, 39_ = 32.30, *P* < 0.001), time coverage (F_3, 39_ = 21.78, *P* < 0.001, **Figure 3D**), and frequency of occurrence (F_3, 39_ = 19.24, *P* < 0.001). Bonferroni-adjusted *post hoc* tests revealed that Map 3 had significantly higher temporal parameter estimates than all other maps (*P* < 0.001). Map 4 had significantly higher GEV compared to Map 1 (*P* = 0.02), higher mean duration compared to Map 1 (*P* < 0.001) and Map 2 (*P* = 0.006), higher time coverage compared to Map 1 (*P* = 0.004) and Map 2 (*P* = 0.01), and higher frequency of occurrence compared to Map 1 (*P* = 0.004) and Map 2 (*P* = 0.01).

For both groups, Map 3 had the highest temporal parameters, followed by Map 4. These maps were the most present and accounted for the majority of the variance in the data. Since these maps were very similar and spatially highly correlated between groups, the inverse solutions for Map 3 and Map 4 were computed for each group at the single subject level and subsequently statistically compared between groups in order to establish group differences in the neural activation patterns of these quasi-similar maps.

### Source Localization of Group Differences

**Figure 4:**
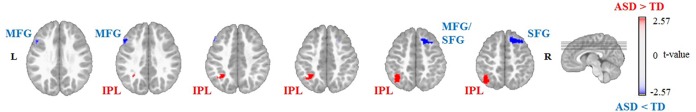
Source localization of group differences for Group Map 3 showing decreased activation of the left and right middle frontal gyrus (MFG) and right superior frontal gyrus (SFG), and increased activation of the left inferior parietal lobule (IPL) in the autism spectrum disorder (ASD) group (n = 14) compared to the typically developing (TD) group (n = 14) during free viewing of “Trotro” cartoons. The t-values of the unpaired t-test thresholded to *P* < 0.01 of the randomization test are plotted. L, left; R, right.

**Figure 5:**
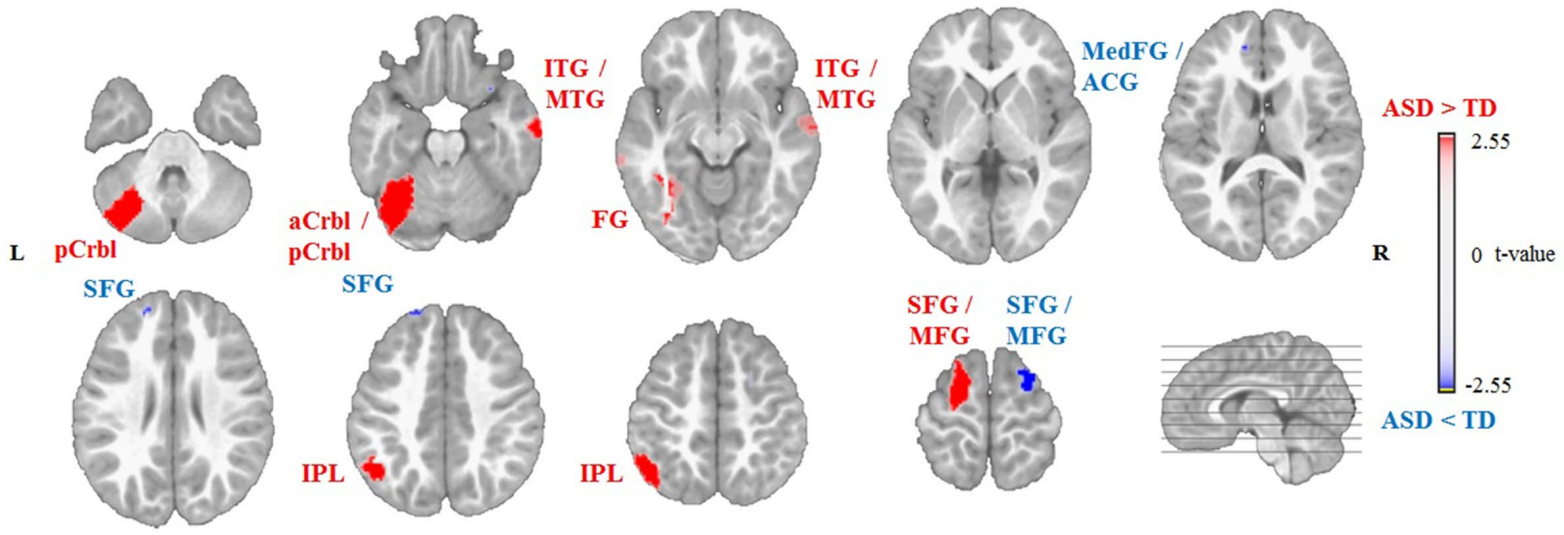
Source localization of group differences for Group Map 4 showing decreased activation of the left medial frontal gyrus (MedFG)/anterior cingulate gyrus (ACG) complex, left and right superior frontal gyrus (SFG), and right middle frontal gyrus (MFG), and increased activation of the left anterior (aCrbl) and posterior cerebellum (pCrbl), left fusiform gyrus (FG), right inferior and middle temporal gyri (ITG/MTG), left inferior parietal lobule (IPL), and left SFG/MFG gyri in the autism spectrum disorder (ASD) group (n = 14) compared to the typically developing (TD) group (n = 14) during free viewing of “Trotro” cartoons. The t-values of the unpaired t-test thresholded to *P* < 0.01 of the randomization test are plotted. L, left; R, right.

Non-parametric permutation tests of the source localization between the two groups showed significant differences in EEG activation (*P* < 0.01) for the sources of Map 3 and Map 4. For Map 3, the ASD group showed decreased activation of the left and right middle frontal gyrus (MFG, Brodmann area (BA) 9 and 8, respectively) and right superior frontal gyrus (SFG, BA 8), and increased activation of the left IPL (BA 40), compared to the TD group (**Table 2**, **Figure 4**). For Map 4, the ASD group exhibited decreased activation of the right MFG (BA 6), left and right SFG (BA 9 and 6, respectively), and left medial frontal gyrus (BA 10)/anterior cingulate gyrus (ACG, BA 42), compared to the TD group. On the other hand, the ASD group showed increased activation of the left anterior cerebellum (culmen), left posterior cerebellum (pyramis, declive), left IPL (BA 40), left FG (BA 37), right middle temporal gyrus (MTG, BA 21), right inferior temporal gyrus (ITG, BA 21), and left SFG/MFG gyri (BA 6) compared to the TD group in Map 4 (**Table 3**, **Figure 5**).

**Table 2:** Source localization of group differences for Group Map 3 showing differences in activation in brain regions, thresholded at P < 0.01, between the autism spectrum disorder (ASD) group (n = 14) compared to the typically developing (TD) group (n = 14) during free viewing of “Trotro” cartoons. The t-values of the unpaired t-test at the corresponding solution point are given.

	Talairach Coordinates	Brain Region	t-value
**ASD < TD**	23, 19, 48	Right superior frontal gyrus, BA 8	–2.90
	23, 18, 42	Right middle frontal gyrus, BA 8	–2.85
	–50, 18, 28	Left middle frontal gyrus, BA 9	–2.81
**ASD > TD**	–37, –54, 45	Left inferior parietal lobule, BA 40	3.26

**Table 3:** Source localization of group differences for Group Map 4 showing differences in activation in brain regions, thresholded at P < 0.01, between the autism spectrum disorder (ASD) group (n = 14) compared to the typically developing group (n = 14) during free viewing of “Trotro” cartoons. The t-values of the unpaired t-test at the corresponding solution point are given.

	Talairach coordinates	Brain region	t-value
**ASD < TD**	30, 0, 56	Right middle frontal gyrus, BA 6	–2.83
	23, 12, 55	Right superior frontal gyrus, BA 6	–3.31
	–16, 51, 27	Left superior frontal gyrus, BA 9	–3.60
	–10, 50, 7	Left middle frontal gyrus, BA 10	–3.12
	–10, 43, 7	Left anterior cingulate gyrus, BA 42	–3.03
**ASD > TD**	–23, 0, 62	Left middle frontal gyrus, BA 6	4.30
	–21, 9, 55	Left superior frontal gyrus, BA 6	3.47
	–52, –54, 42	Left inferior parietal lobule, BA 40	4.34
	64, –17, –8	Right middle temporal gyrus, BA 21	2.78
	64, –10, –14	Right inferior temporal gyrus, BA 21	2.99
	–30, –50, –6	Left fusiform gyrus, BA 37	3.04
	–23, –58, –24	Left anterior cerebellum, culmen	3.46
	–37, –71, –17	Left posterior cerebellum, declive	3.41
	–23, –71, –29	Left posterior cerebellum, pyramis	2.80

### Subgroup Analysis With Respect to Gaze Pattern

Simultaneously to EEG data acquisition, high-resolution eye-tracking was performed on all participants. From the TD group, a normative gaze distribution was obtained by applying a kernel density distribution estimation on gaze data at each frame of the Trotro movies. Following this, the probability of proximity to this pre-established “norm” was computed frame-by-frame for each participant with ASD. According to this analysis, the ASD group was mean-split into two subgroups according to the similarity of their average gaze with the TD group. The first subgroup had gaze patterns more similar to the “norm,” referred to as the CS ASD subgroup, and the second subgroup had gaze patterns dissimilar to the “norm,” referred to as the CD ASD subgroup (**Figure 1**). Given the small sample size of the ASD subgroups (n = 8 and n = 6, respectively), the separate analysis of the source patterns comparing the subgroups with the TD group is reported in the **Supplementary Material** only.

## Discussion

The current study used high-density EEG and eye-tracking to investigate differences in neural activation in the brains of children with ASD and their TD peers during free viewing of dynamic semi-naturalistic stimuli containing social interactions. Using an EEG microstate approach, we identified four dominant topographic maps that had significantly different temporal dynamics from one another for each group and were very similar and spatially highly correlated between the groups. Two of the four microstate maps (Maps 3 and 4) that had the highest temporal parameters in both groups were compared in the inverse space to reveal group differences in neural activation during viewing of the Trotro cartoons. Notably, children with ASD showed decreased prefrontal and cingulate activation, impaired activation of the premotor cortex, and increased activation of parietal, temporal, occipital, and cerebellar regions, compared to their TD peers. To the best of our knowledge, only one recent study with ASD participants (aged between 5 and 18 years) and TD participants (aged between 5 and 15 years) using an EEG microstate approach has been published. Results from this study revealed the presence of four dominant topographic maps in the resting-state EEG, with statistically significant differences between the individuals with ASD and their TD peers among the temporal parameters evaluated. However, only temporal parameters were compared, and no source localization was performed (61).

Subsequent analyses comparing subgroups within the ASD group with the TD group (see **Supplementary Material**) revealed decreased activation of the prefrontal, premotor, and cingulate regions in CD ASD but not CS ASD, compared to TD children. There was a contrasting increase in activation in the premotor cortex of CS ASD children, compared to TD children. Increased temporal activation was found only in the CD ASD subgroup, whereas increased activation of the cerebellum and parietal cortex was found in both ASD subgroups in comparison to the TD group. Most of the brain regions reported in the current study, where group differences in neural activity were present, have roles in social and non-social executive functioning (3, 10, 11). Given the social nature of the semi-naturalistic stimuli presented, the results are interpreted in the context of social cognition.

### The Prefrontal, Premotor, and Cingulate Cortices

We reported decreased activation within the frontal cortex of children with ASD compared to TD children. For Map 3, this decreased activation was found in the left dorsolateral prefrontal cortex (DLPFC, within MFG, BA 9) and in the right premotor cortex (within the SFG/MFG, BA 8). Similarly, for Map 4, children with ASD exhibited decreased activation of the left DLPFC (within the SFG, BA 9) and the right premotor cortex (within the MFG and SFG, BA 6). Additionally, for Map 4, the ASD group showed decreased activation of the left dorsal medial prefrontal cortex (DMPFC, within the medial frontal gyrus, BA 10), compared to children with TD. These results would suggest the presence of alterations within these areas in children with ASD while viewing the social scenes. Recently, using directed functional connectivity analyses, based on electrical source imaging, we found altered connectivity in the theta frequency band within several of the frontal and the cingulate regions while children with ASD were watching movies of biological motion (28). This would suggest that alterations in the spatio-temporal EEG microstates and the directed functional connectivity are already present at early developmental stages in ASD.

The DMPFC is involved in self-referential activities (62), considering the mental states of others, and “theory of mind” (63, 64), and has a general role in social cognitive processing (65). The DMPFC is a region likely to develop abnormally in ASD, possibly resulting in individuals with ASD showing atypical activation of this region on social–cognitive tasks (13). Children with ASD have been shown to have a disturbance of dopaminergic activity within this region (66), and adults with ASD have been shown to have decreased grey matter density within the DMPFC (67). In addition to decreased activation within the DMPFC in Map 4, we reported reduced activation of the ACC (within the ACG, BA 42), compared to TD children. This finding is in line with the existing literature; an activation likelihood estimation meta-analysis of 50 neuroimaging studies of social cognition in children and adults with ASD highlighted the cingulate cortex as one of the main regions with decreased activity compared to TD children and healthy adults (12). The ACC is an important region within the “social brain” and is involved in goal-directed behaviors, including control of saccadic movements during visual orienting (68). Structurally, children and adults with ASD have been reported to have reduced grey matter (15) and white matter (69) volumes within the ACC. Impairments in the functioning of the ACC and DMPFC, together, are thought to be involved in atypical social orienting, and social cognition and may be a substrate for this in ASD (13, 68).

The DLPFC plays an important role in higher cognition, including executive functioning, and has reciprocal connections with the ACC and with regions involved in motor control, such as the premotor cortex and basal ganglia, and in higher-order sensory processing, such as the parietal and temporal cortices (70). The premotor cortex is involved in the selection of movements and in encoding the intention to perform certain movements based on external visual cues (71). Our sample of children with ASD exhibited decreased activation of the left and right premotor cortex (BA 6), but they also exhibited increased activation in the left SFG/MFG gyri (BA 6), compared to the TD group, suggesting a general dysfunction in the premotor cortex of children with ASD. The reduction in activation of the DLPFC and ACC, coupled with dysfunctional activation of the premotor cortex, may suggest an impaired ability to appropriately process the dynamic motion stimuli presented by the Trotro cartoons in our sample of children with ASD. Furthermore, decreased activation of both the ACC and DMPFC in these children during free viewing of the Trotro cartoons suggests a deficit in detecting and/or understanding the social content of the cartoon movies.

The subgroup analysis (see **Supplementary Material**) indicated that impaired activation of the premotor cortex was present in both subgroups in opposing directions; while the CS ASD subgroup showed increased activation of the premotor cortex, the CD ASD subgroup exhibited decreased activation of this region, compared to TD children. This finding suggests that increasing activation of the premotor cortex may be part of the mechanism by which CS ASD children establish gaze patterns that more closely follow moving targets on the screen, in a similar manner to TD children.

### The Parietal Cortex

In contrast to the reduced activation of the frontal, premotor, and cingulate cortices, increased activation of the left IPL was observed in our group of children with ASD compared to those with TD, regardless of their gaze patterns. A magnetoencephalography (MEG) study performed during rest using a source-space approach found altered coherence within parietal regions; however, the ASD group in this study only included adolescents (72). The IPL is an integral part of the mirror neuron system; it is connected with the ventral premotor cortex and plays a fundamental role in processing visual and somatosensory information (73, 74). The mirror mechanism is involved in understanding others’ actions and intentions and is located in the same areas that are involved in goal-directed actions, the parieto-frontal network. In brief, the mirror mechanism entails that the motor system is involved not only in producing movements but also in cognitive functions such as observing motor behaviors, and these observations consequently result in motor activation, as if the observer is mirroring the action being executed by someone else (75). The “direct matching hypothesis” maintains that activation of the mirror neuron system upon observing an action is essential to understanding the goal of that action (76). The parieto-frontal circuit involved in the mirror mechanism is thought to be dysfunctional in ASD (16, 75, 77, 78). Our study shows increased activation of the IPL in children with ASD compared to those with TD. A recent activation likelihood estimation meta-analysis reports stronger effects in the IPL of individuals with ASD compared to TD individuals (78). Therefore, our finding of increased IPL activation in children with ASD, regardless of their gaze patterns, may suggest a dysfunction of the mirror neuron system upon observing actions of the animated Trotro donkey characters, which is evident from a young age.

### The Cerebellum

The cerebellum has long been recognized for its fundamental role in movement coordination and balance; however, it is also considerably involved in a variety of other functions including cognition, emotion, and perception, such as motion perception [for a review, see Baumann et al. (79)]. Moreover, the cerebellum has an important role in oculomotor control and has connections to regions within the prefrontal and parietal cortices, which are involved in visuospatial attention (80). Thus, it is thought to be essentially involved in controlling covert visual attention (80–82).

Although we found that our group of children with ASD, specifically the CD ASD subgroup (see **Supplementary Material**), showed increased activation within the left anterior and posterior cerebellum, compared to the TD group, these findings should be interpreted with care. Using MEG and an object recognition task, Peiker and colleagues (83) have shown that perceptual integration deficits observed in adults with ASD are related to alterations in the connectivity between the left cerebellum and right posterior superior temporal sulcus. Moreover, increased motor activation of the cerebellum but decreased cerebellar attention activation have been previously reported in adults with ASD during motor and attention tasks, respectively (83). It is possible that our ASD group may suffer from more pronounced cerebellar impairments than TD children and that this has a functional implication on their ability to control their eye movements through the cerebellum, preventing them from assembling visual details into an entire and integrated percept. Over-activity of the cerebellum in the ASD group may suggest an impairment in the ability of the cerebellum to maintain the accuracy of saccades onto visual targets within the Trotro cartoon. Alternatively, as reduced processing of the irrelevant context has been previously reported in older children and adults with ASD (85), increased activation of the cerebellum may be due to the lack of ability of the ASD group to distinguish the socially relevant from irrelevant information in the cartoons.

### The Anterior Temporal Lobe

Anterior portions of the fusiform, inferior, and middle temporal gyri form part of the anterior temporal lobe (86), which is thought to play an important role in storing and retrieving social knowledge (87). These regions form part of the social brain, which has been reported to be altered in ASD. Our results showed that children with ASD exhibited increased activation of the right MTG (BA 21), ITG (BA 21), and left FG (BA 37), compared to TD children. However, the subgroup analysis (see **Supplementary Material**) indicated that this increase in activation might only be present in the CD ASD and not CS ASD children, when compared to TD children. Thus, increased activation of these regions may reflect an impairment in the function of the anterior temporal lobule to appropriately process social information from the cartoon stimuli, possibly explaining the deviance of gaze of the CD ASD children from socially relevant parts of the cartoon scenes.

### Limitations

This study has several limitations. The sample sizes are small (ASD and TD, n = 14), mainly due to the difficulty of collecting EEG data that are free from movement artifacts from a pediatric population. Owing to the small sample size, the subgroup analysis that took gaze behavior into account was underpowered (CS ASD n = 8, CD ASD n = 6). The results of the subgroup analysis (see **Supplementary Material**) are insightful and interesting; however, they need to be consolidated by future studies with larger sample sizes.

While direct evidence that scalp EEG can capture subcortical signals has recently been produced (88), whether EEG can detect subcortical signals is still a matter of debate. Therefore, it is important to consolidate the present findings, particularly those in deeper brain regions, with future studies using methodologies of higher spatial resolution such as functional magnetic resonance imaging.

## Conclusions

A combination of frontal and parietal processing is thought to be optimal for understanding social situations; however, these areas, amongst several others, have been shown to be dysfunctional in ASD. Our findings suggest that from a young age, children with ASD, especially those with gaze patterns that diverged from the gaze patterns of TD children, exhibited abnormalities in neural activation during free viewing of dynamic social stimuli, including reduced activation of frontal and cingulate regions and increased activation of inferior parietal, temporal, and cerebellar regions. These results suggest that children with ASD, particularly those with CD gaze patterns, process the visual stimuli differently and fail to detect the social information. Eye-tracking and high-density EEG may be a promising combination that could aid in diagnostic differentiation of different ASD subtypes in the future, possibly leading to more targeted treatment interventions.

## Data Availability

The datasets for this manuscript are not publicly available because analysis of these data is ongoing as part of a longitudinal study, and results are expected to be published in the future. When all data has been published, requests to access the datasets should be directed to Dr Marie Schaer, marie.schaer@unige.ch.

## Author Contributions

RKJ conducted data collection and analysis and the writing of this manuscript, and is the primary and corresponding author. TAR was involved in experimental design, data collection, and analysis, and contributed feedback on the written manuscript. NK conducted participant recruitment and participated in data collection and in the design of the novel eye-tracking analysis method used in the current study. HFS was involved in data collection and contributed feedback on the written manuscript. MF conducted participant recruitment and participated in experimental design and data collection. AC and MIT provided intellectual input on EEG data analysis. CMM was involved in experimental design and provided intellectual input on all aspects of the EEG data analysis and the written manuscript. MS is the principal investigator and was involved in experimental design, participant recruitment, and providing intellectual input on data analysis and feedback on the written manuscript. All authors read and approved the final manuscript.

## Funding

This work was supported by the National Center of Competence in Research (NCCR) “SYNAPSY—The Synaptic Bases of Mental Diseases” financed by the Swiss National Science Foundation (SNF, grant no. 51AU40_125759), SNF grant no. 163859 to MS, and grant no. 320030_184677 to CM; private funding from Fondation Pole Autisme (http://www.pole-autisme.ch); and a Marie Curie fellowship to RJ, which received funding from the European Union Seventh Framework Programme (FP7/2007-2013) under grant agreement no. 267171.

## Conflict of Interest Statement

The authors declare that the research was conducted in the absence of any commercial or financial relationships that could be construed as a potential conflict of interest.

## Abbreviations

ANOVA, Analysis of variance; ACC, anterior cingulate cortex; ACG, anterior cingulate gyrus; ADOS, Autism Diagnostic Observation Schedule; ASD, autism spectrum disorder; BA, Brodmann area; CD, control-dissimilar; CS, control-similar; DLPFC, dorsolateral prefrontal cortex; DMPFC, dorsal medial prefrontal cortex; EEG, electroencephalography; FG, fusiform gyrus; GEV, global explained variance; GFP, global field power; IPL, inferior parietal lobule; ITG, inferior temporal gyrus; LAURA, Local Autoregressive Average; MFG, middle frontal gyrus; MTG, middle temporal gyrus; SFG, superior frontal gyrus; TD, typically developing.

## Supplementary Material: Subgroup Analysis according to Gaze Patterns

### Method: Eye-tracking Data Collection and Analysis

Simultaneously to EEG data acquisition, high-resolution eye-tracking was performed on all participants using a Tobii TX300 system (Tobii® Technology, Sweden) with a sampling rate of 300 Hz. A data-driven method developed by our group was used to define age-appropriate dynamic “norms” of visual exploration of the complex social scenes presented in the “Trotro” cartoon movies (89). For the calculation of the normative gaze distribution, gaze data from the 14 TD children in the EEG analysis, as well as an additional 12 TD children who did not have EEG data but had good eye-tracking data, were combined resulting in a larger sample size of 26 TD children (mean age 3.4 ± 1.2 years), which was appropriate for defining the “norm”. Normative gaze distribution was obtained by applying the kernel density distribution estimation on gaze data at each frame of the “Trotro” movies (90). Following this, the probability of proximity to this pre-established “norm” was computed frame-by-frame for each participant with ASD from our sample. For each participant with ASD, these values were averaged across the four “Trotro” cartoon conditions to obtain a single mean value that described the probability of proximity for that participant to the average gaze pattern of the TD group. Higher values of this measure represented a visual exploration pattern that was similar to that of the TD group, while lower values represented a pattern that was dissimilar or further away from that of the TD group. The gaze patterns differed for each child in the ASD group; some following a pattern close to that of the TD children and some following a completely different pattern. Therefore, a mean-split of the average proximity to the “norm” was used for the ASD group in order to establish two subgroups which did not significantly differ in mean age (t-test, P = 0.86) or ADOS severity scores (t-test, P = 0.30). The first subgroup had gaze patterns more similar to the “norm”, referred to as the control-similar (CS) ASD subgroup (n = 8, mean age 3.3 ± 0.8 years, mean ADOS severity 6.9 ± 1.9), and the second subgroup had gaze patterns dissimilar to the “norm”, referred to as the control-dissimilar (CD) ASD subgroup (n = 6, mean age 3.3 ± 0.8 years, mean ADOS severity 8.0 ± 2.0) (**Figure 1**). The sources of the microstate maps 3 and 4 of these subgroups were then separately compared to the TD group by unpaired randomization tests.

### Results: Source Localization of Subgroup Differences

#### Control-Similar (CS) ASD vs. TD

In comparison to the TD group, the CS ASD group exhibited increased activation of the left IPL (BA 40) for Map 3 (**Supplementary Figure 1**) and Map 4 (**Supplementary Figure 2**), and of the left posterior cerebellum (declive), and left SFG/MFG (BA 6) for Map 4 only.

#### Control-Dissimilar (CD) ASD vs. TD

For Map 3, the CD ASD group showed decreased activation of the left MFG (BA 9) and right SFG (BA 8), and increased activation of the right MTG (BA 21), right ITG (BA 21), right FG (BA 19), right precentral gyrus (BA 6), and left IPL (BA 40), compared to the TD group (**Supplementary Figure 3**). For Map 4, the CD ASD group showed decreased activation of the left medial frontal gyrus (BA 10)/ACG (BA 32), left SFG (BA 9) and right MFG (BA 6), and increased activation of the left and right anterior cerebellum (culmen), left posterior cerebellum (declive), right posterior cerebellar tonsil, right MTG (BA 21), right ITG (BA 21), left and right FG (BA 37) and left IPL (BA 40), in comparison to the TD group (**Supplementary Figure 4**).

Supplementary Figure 1 Source localisation of group differences for Group Map 3 showing increased activation of the left inferior parietal lobule (IPL) in the control-similar (CS) autism spectrum disorder (ASD) group (n = 8) compared to the typically developing (TD) group (n = 14) during free viewing of “Trotro” cartoons. The t-values of the unpaired t-test thresholded to *P*< 0.01 of the randomization test are plotted. L, Left; R, Right.

Supplementary Figure 2 Source localisation of group differences for Group Map 4 showing increased activation of the left posterior cerebellum (pCrbl), left inferior parietal lobule (IPL), and left superior and middle frontal gyri (SFG/MFG) in the control-similar (CS) autism spectrum disorder (ASD) group (n = 8) compared to the typically developing (TD) group (n = 14) during free viewing of “Trotro” cartoons. The t-values of the unpaired t-test thresholded to *P*< 0.01 of the randomization test are plotted. L, Left; R, Right.

Supplementary Figure 3 Source localisation of group differences for Group Map 3 showing decreased activation of the left middle frontal gyrus (MFG) and right superior frontal gyrus (SFG), and increased activation of right inferior temporal gyrus (ITG), middle temporal gyrus (MTG), fusiform gyrus (FG), precentral gyrus (PCG), and left inferior parietal lobule (IPL) in the control-dissimilar (CD) autism spectrum disorder (ASD) group (n = 6) compared to the typically developing (TD) group (n = 14) during free viewing of “Trotro” cartoons. The t-values of the unpaired t-test thresholded to *P*< 0.01 of the randomization test are plotted. L, Left; R, Right.

Supplementary Figure 4 Source localisation of group differences for Group Map 4 showing decreased activation of the left medial frontal gyrus (MedFG)/anterior cingulate gyrus (ACG), left superior frontal gyrus (SFG) and right middle frontal gyrus (MFG), and increased activation of the left and right anterior (aCrbl) and posterior cerebellum (pCrbl), right middle temporal gyrus (MTG), right inferior temporal gyrus (ITG), left and right fusiform gyrus (FG), and left inferior parietal lobule (IPL) in the control-dissimilar (CD) autism spectrum disorder (ASD) group (n = 6) compared to the typically developing (TD) group (n = 14) during free viewing of “Trotro” cartoons. The t-values of the unpaired t-test thresholded to *P*< 0.01 of the randomization test are plotted. L, Left; R, Right.
