# Supplementary figures and images for: Neural Processing of Dynamic Animated Social Interactions in Young Children With Autism Spectrum Disorder: A High-Density Electroencephalography Study
Source: Front Psychiatry. 2019 Aug 22;10:582. doi: 10.3389/fpsyt.2019.00582 (PMC6714589; doi:10.3389/fpsyt.2019.00582)

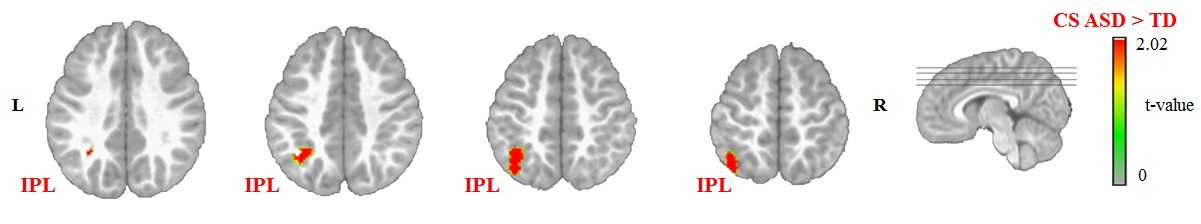

Supplement: Supplementary Figure 1 — Source localisation of group differences for Group Map 3 showing increased activation of the left inferior parietal lobule (IPL) in the control-similar (CS) autism spectrum disorder (ASD) group (n = 8) compared to the typically developing (TD) group (n = 14) during free viewing of “Trotro” cartoons. The t-values of the unpaired t-test thresholded to P< 0.01 of the randomization test are plotted. L, Left; R, Right. [file Image_1.jpg]

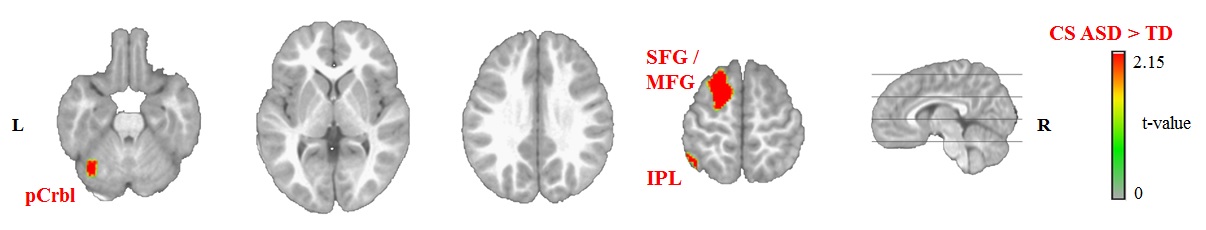

Supplement: Supplementary Figure 2 — Source localisation of group differences for Group Map 4 showing increased activation of the left posterior cerebellum (pCrbl), left inferior parietal lobule (IPL), and left superior and middle frontal gyri (SFG/MFG) in the control-similar (CS) autism spectrum disorder (ASD) group (n = 8) compared to the typically developing (TD) group (n = 14) during free viewing of “Trotro” cartoons. The t-values of the unpaired t-test thresholded to P< 0.01 of the randomization test are plotted. L, Left; R, Right. [file Image_2.jpg]

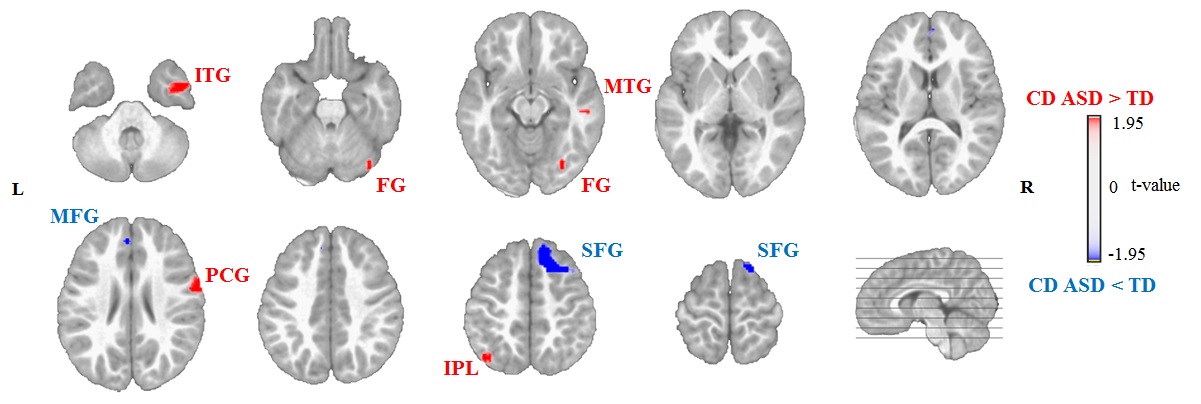

Supplement: Supplementary Figure 3 — Source localisation of group differences for Group Map 3 showing decreased activation of the left middle frontal gyrus (MFG) and right superior frontal gyrus (SFG), and increased activation of right inferior temporal gyrus (ITG), middle temporal gyrus (MTG), fusiform gyrus (FG), precentral gyrus (PCG), and left inferior parietal lobule (IPL) in the control-dissimilar (CD) autism spectrum disorder (ASD) group (n = 6) compared to the typically developing (TD) group (n = 14) during free viewing of “Trotro” cartoons. The t-values of the unpaired t-test thresholded to P< 0.01 of the randomization test are plotted. L, Left; R, Right. [file Image_3.jpg]

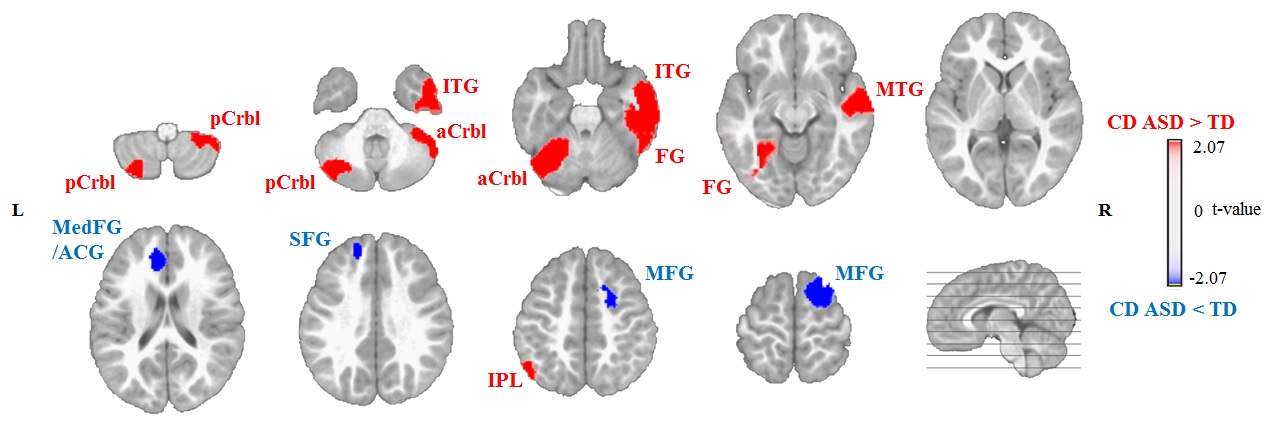

Supplement: Supplementary Figure 4 — Source localisation of group differences for Group Map 4 showing decreased activation of the left medial frontal gyrus (MedFG)/anterior cingulate gyrus (ACG), left superior frontal gyrus (SFG) and right middle frontal gyrus (MFG), and increased activation of the left and right anterior (aCrbl) and posterior cerebellum (pCrbl), right middle temporal gyrus (MTG), right inferior temporal gyrus (ITG), left and right fusiform gyrus (FG), and left inferior parietal lobule (IPL) in the control-dissimilar (CD) autism spectrum disorder (ASD) group (n = 6) compared to the typically developing (TD) group (n = 14) during free viewing of “Trotro” cartoons. The t-values of the unpaired t-test thresholded to P< 0.01 of the randomization test are plotted. L, Left; R, Right. [file Image_4.jpg]
